# Recent advances of PET imaging in clinical radiation oncology

**DOI:** 10.1186/s13014-020-01519-1

**Published:** 2020-04-21

**Authors:** M. Unterrainer, C. Eze, H. Ilhan, S. Marschner, O. Roengvoraphoj, N. S. Schmidt-Hegemann, F. Walter, W. G. Kunz, P. Munck af Rosenschöld, R. Jeraj, N. L. Albert, A. L. Grosu, M. Niyazi, P. Bartenstein, C. Belka

**Affiliations:** 1 Department of Nuclear Medicine, University Hospital, LMU Munich, Marchioninistr. 15, 81377 Munich, Germany; 2 Department of Radiology, University Hospital, LMU Munich, Marchioninistr. 15, 81377 Munich, Germany; 3 German Cancer Consortium (DKTK), partner site Munich; and German Cancer Research Center (DKFZ), Heidelberg, Germany; 4 Department of Radiation Oncology, University Hospital, LMU Munich, Munich, Germany; 5 Radiation Physics, Department of Hematology, Oncology and Radiation Physics, Skåne University Hospital, and Lund University, Lund, Sweden; 6 Department of Medical Physics, School of Medicine and Public Health, University of Wisconsin-Madison, Madison, USA; 7 Department of Radiation Oncology, Medical Center - University of Freiburg, Faculty of Medicine, University of Freiburg, Freiburg, Germany; 8 German Cancer Consortium (DKTK), partner Site Freiburg, Freiburg, Germany

**Keywords:** PET, Radiation oncology, Neuro-oncology, Head & neck cancer, Lung cancer, Prostate cancer, GI malignancies

## Abstract

Radiotherapy and radiation oncology play a key role in the clinical management of patients suffering from oncological diseases. In clinical routine, anatomic imaging such as contrast-enhanced CT and MRI are widely available and are usually used to improve the target volume delineation for subsequent radiotherapy. Moreover, these modalities are also used for treatment monitoring after radiotherapy. However, some diagnostic questions cannot be sufficiently addressed by the mere use standard morphological imaging. Therefore, positron emission tomography (PET) imaging gains increasing clinical significance in the management of oncological patients undergoing radiotherapy, as PET allows the visualization and quantification of tumoral features on a molecular level beyond the mere morphological extent shown by conventional imaging, such as tumor metabolism or receptor expression. The tumor metabolism or receptor expression information derived from PET can be used as tool for visualization of tumor extent, for assessing response during and after therapy, for prediction of patterns of failure and for definition of the volume in need of dose-escalation. This review focuses on recent and current advances of PET imaging within the field of clinical radiotherapy / radiation oncology in several oncological entities (neuro-oncology, head & neck cancer, lung cancer, gastrointestinal tumors and prostate cancer) with particular emphasis on radiotherapy planning, response assessment after radiotherapy and prognostication.

## Introduction

Radiotherapy plays a key role in the clinical management of patients suffering from oncological diseases, as approximately half of cancer patients directly benefit from individual radiotherapy during their disease course. In this disease course, radiotherapy can be applied as sole treatment or as a comprehensive treatment in combination with systemic treatments such as chemotherapy or local treatments such as surgery [1]. This high clinical significance for the treatment of oncological diseases is reached and maintained by the fast technological innovation and improvements that were introduced and subsequently established in clinical routine over the last decades [2], e.g. intensity-modulated radiation therapy (IMRT) has evolved as a widely used clinical treatment modality in many countries [3].

Anatomic imaging such as contrast-enhanced CT and MRI are widely available and are usually used to delineate the target volume for the subsequent radiotherapy. However, in the clinical routine in radiation oncology, diagnostic issues arise that cannot be sufficiently addressed by standard morphologic imaging. In particular, the delineation of viable tumor tissue can be challenging, especially in patients with local pretreatment such as surgery. Moreover, treatment response assessment with conventional morphological imaging is partly unable to correctly differentiate early relapse from radiation induced changes or inflammation, e.g. in neuro-oncology [4]. Therefore, positron emission tomography (PET) imaging gains increasing clinical significance in the management of oncological patients undergoing radiotherapy, as PET allows the visualization and quantification of tumoral features on a molecular level beyond the mere morphological extent on conventional imaging, such as tumor metabolism or receptor expression. ^18^F-FDG, a glucose analogue, is the most commonly applied ligand for oncological PET imaging [5] due to its proven utility and its generally increasing availability. Beyond the visualization of glucose metabolism, other tumor characteristics can be targeted and visualized by PET imaging. In this regard, e.g. PET with prostate-specific membrane antigen (PSMA) ligands are of high clinical and scientific interest for advanced imaging of patients suffering from prostate cancer [6]. The tumor metabolism or receptor expression information has allowed for use as a tool for (a) visualization of tumor extent, for (b) assessing response during and (c) after therapy, for (d) prediction of patterns of failure and for (e) definition of the volume in need of dose-escalation. Where, (e) sometimes has been referred to as “dose-painting” [7], although the idea is older [8] and the practice of escalation of the PET-avid volumes has been in long use for the treatment of e.g. head neck cancer.

This review describes the recent advances of PET imaging within the field of clinical radiotherapy / radiation oncology in several oncological diseases (neuro-oncology, head & neck cancer, lung cancer, gastrointestinal tumors and prostate cancer) with particular emphasis on radiotherapy planning, but also on treatment response evaluation and prognostication. Moreover, recent advances in PET imaging itself are reviewed with special emphasis on the potential applicability on clinical settings in radiotherapy / radiation oncology.

## Neuro-oncology

PET is widely applied in the field of neuro-oncology as complementary imaging modality in addition to MRI [9]. Its use may be derived from the answers to several key questions: 1) How to optimally define the radiotherapeutic target volume or delineate the extent of disease before surgical resection, 2) is it possible to derive prognostic value from molecular imaging, and 3) how to distinguish treatment effect from true progression.

When considering the wide field of primary CNS tumors, the entity of glioma is reported on by the PET task force of the Response Assessment in Neuro-Oncology (RANO) working group [10]. This task force clearly derives evidence from published studies with validated PET findings (either by histopathology or clinical course) in the setting of diagnosis, biopsy, surgery, radiotherapy and response assessment, and shows superiority of amino acid PET such as ^18^F-FET or ^11^C-MET PET [11] over ^18^F-FDG PET [10, 12]. Specifically, ^18^F-FET has been shown to predict prognosis [13, 14], to enable improved target delineation [15–17] to assess treatment response [10]. Recurrence pattern analyses have substantiated the role of amino acid PET in identifying aggressive parts and the potential of targeting these regions [18–21]. In a recent study, the combination of ^18^F-FET-PET and T1w MRI was shown to carry the most information for prediction of patterns of failure following chemo-radiation therapy of glioblastoma patients [22]. In the US, ^18^F-DOPA is a widely used tracer and it was shown to provide additional clinical information [23], which could also be validated histopathologically [24]. A variety of data exists on other tracers as described in Table 1 [28]. One potential target of interest for brain tumor imaging is the 18 kDa translocator protein (TSPO), as known in neurodegenerative research, with remarkable overexpression in glioblastoma patients, whereas further studies have to further elucidate the contribution of neuro-inflammatory component within the signal obtained in TSPO PET [29–31]. In this regard, the potential influence of this new modality on radiotherapy approaches has to be validated. In sum, especially amino acid tracers are applied for radiotherapy planning in clinical routine of glioma patients [9, 15, 20], but also for the differentiation of viable tumor and recurrent / progressive disease after initial radiotherapy [4, 32, 33], as recently emphasized by the PET RANO group [10].

**Table 1:** Different tumors and tracers in neuro-oncology for different indications: target delineation (TD), prognostication (P), distinguishing between progressive disease and pseudoprogression (TR)

Tumor entity	Tracers	Indication	Comment
**Glioma**	^18^F-FET	TD/P/TR	Valuable as longer halftime compared to ^11^C-MET, high diagnostic accuracy with histopathological validation; ongoing trials to confirm clinical benefit, e. g. GLIAA [25]
	^18^F-DOPA	TD/P/TR	Studies on prognostic relevance and histopathological validation available, e.g. [24, 26], mainly used in the US
	^11^C-MET	TD/P	Studies on prognostic relevance and histopathological validation available. Aiding in target delineation.
	TSPO ligands	None	Investigational, no histopathological validation studies (ongoing)
**Meningioma**	^68^Ga-DOTATOC	TD	Aiding in target delineation or surgical approach, especially when located at the skull base
	^68^Ga-DOTATATE	TD	SUV cutoff histologically validated, no relevant data available on response
**Brain metastasis**	^18^F-FET	TR	Differentiation pseudoprogression/radiation necrosis vs. tumor recurrence
**CNS lymphoma**	^18^F-FDG	None	Tumor metabolism, response assessment [27]

**Fig. 1:**
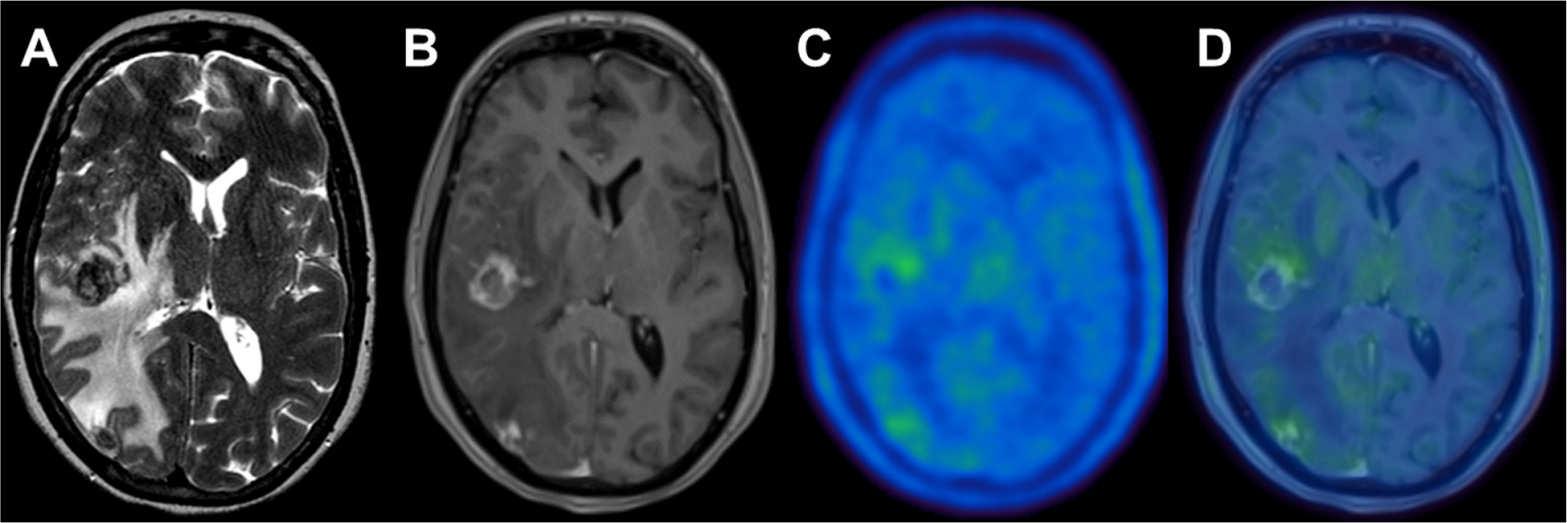
A 54 years-old female patient with extensive edema on T2 MRI (**a**) and new contrast enhancing lesions at the temporal and occipital lobe (**b**) after undergoing stereotactic radiosurgery for brain metastases from malignant melanoma at both sites. MRI findings were suggestive for tumor recurrence, whereas only a faint uptake on ^18^F-FET PET (**c**) and fused PET/MRI (**d**) was seen in both lesions, a finding typical for radiation necrosis. Radiation necrosis was subsequently confirmed by histopathology

In analogy to primary brain tumors, brain metastases can also be visualized by PET [34]. Although its value for imaging prior to radiotherapy remains unclear, PET imaging, especially with radiolabeled amino acids, has evolved as complementary imaging tool for the differentiation of true progression from pseudoprogression, e.g. after radiotherapy [16, 35–37], see Fig. 1. Therefore, the use of PET in brain metastases was also recently recommended by the PET RANO group [34].

**Fig. 2:**
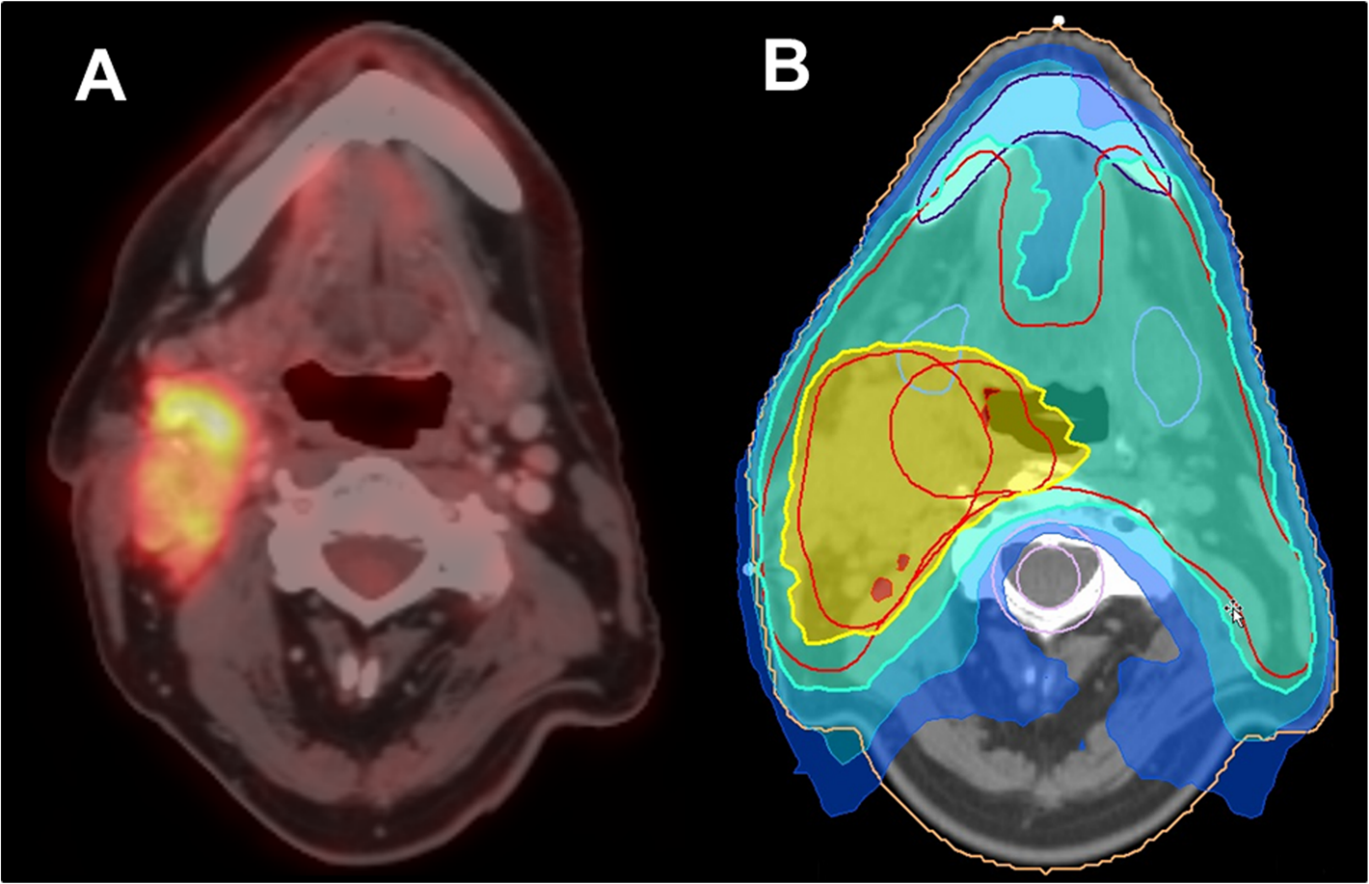
Patient with HPV-positive squamous cell carcinoma of the right tonsil (T1 cN3 M0), who underwent ^18^F-FDG-PET/CT for staging prior to radiotherapy and subsequent inclusion of PET-positive tumor masses and lymph nodes in radiotherapy planning

Compared to glioma and brain metastases, meningioma as extraaxial tumor is even more common. Beyond MRI, PET ligands targeting the somatostatin receptor (SSR) such as ^68^Ga-DOTATOC and ^68^Ga-DOTATATE are used in clinical routine [38, 39] and have been established for surgical guidance [40] or target volume definition [41, 42] due to the high expression of SSR in meningioma tissue. Specifically, this imaging modality is of help in meningiomas at the skull base, where extraforaminal extension or osseous infiltration may be expected [43] or in case of suspected residual or recurrent tissue after initial therapy [40]. Some other reports on amino acid PET are available as well, however, in the light of SSR-ligands, these tracers are not widely used in clinical routine for meningioma imaging [44]. Beyond in CNS lymphoma [45], ^18^F-FDG PET is not recommended by the PET RANO group for most primary brain tumors [10, 34, 46], mainly due to high background activity of the normal brain.

## Head and neck cancer

Head and neck cancers (HNC) consist of a wide range of tumor entities such as squamous cell cancer, salivary tumors or nasopharyngeal carcinomas. Diagnosis and treatment of the group of HNC is a complex and multidisciplinary approach. PET/CT provides insights into tumor biology and tissue metabolism and has an unprecedented accuracy in unmasking nodal metastases or tumor extensions. At the current state, most of the available data for PET imaging in HNC is validated for head and neck squamous cell cancer. PET/CT facilitates contouring for (chemo-) radiotherapy (CRT) and it significantly influences dose painting in radiation planning. In about 25% of patients with disease of unknown primary, location is revealed by ^18^F-FDG-PET/CT [47–52].

Since HNC represents a very heterogeneous disease, there is great interest in finding prognostic markers for risk stratification. For primary staging, the use of PET/CT leads to a change of about 10% in every TNM category and similarly, a major change in treatment strategy in about 10% of patients [53]. This is crucial, knowing that survival decreases by 40–50% in patients with positive lymph nodes [48, 49]. Moreover, first data suggests that the use of PET/CT for radiation planning could significantly improve the local tumor control, regional control and even survival [54]. The maximal standardized value (SUV_max_) of the primary tumor and total lesion glycolysis (TLG) of the largest node on ^18^F-FDG-PET are PET derived parameters that can be used as predictors of therapeutic failure and vice versa [55, 56]. Due to the progress of artificial intelligence and deep learning, radiomics is increasingly becoming the focus of research [57, 58]. Here, radiomic texture parameters such as homogeneity and the sphericity described by Fujima et al. showed high association with the individual clinical course [57]. Moreover, e.g. low-intensity long-run emphasis (LILRE) performed before therapy was stated as a significant predictor of local control after CRT [59]. For a patient example please see Fig. 2.

Signatures similar to radiomics build derived from ^18^F-FDG-PET and contrast-enhanced CT could even predict hypoxic areas of HNC [60], which is an important finding, as tumoral hypoxia is highly associated with an aggressive tumor phenotype that alters gene expression to promote survival in a hostile environment, which unfortunately causes a certain degree of therapeutic resistance [61]. Therefore, the identification of radiation-resistant tumor subvolumes may allow for intensified or hypoxia-modified treatment as well as stratification of patients [62]. Since the first application of hypoxia imaging with PET-ligands in 1981, various tracers like ^18^F-FETNIM, ^18^F-HX4 and ^18^F-FMISO have been evaluated in cancer patients for detecting hypoxic hot spots [61]. Radiation dose escalation up to 77Gy to hypoxic areas detected by hypoxia-specific PET led to better local control without added toxicity, even, when hypoxia imaging used as imaging modality for response assessment during therapy, persistent tumoral hypoxia predicts poorer outcome [61, 63–67]. On the other hand, when comparing different tracers intra-individually, it could be shown that the detected hypoxic areas are often already covered by ^18^F-FDG-avid areas or are in close proximity. Hence, hypoxic areas are mostly included, if radiotherapy is escalated to ^18^F-FDG-avid (sub-) volumes. However, this might lead to larger irradiated volumes and, as a consequence, potentially might result in a higher rate of side effects [68–70]. In sum, hypoxia imaging allows additional insights in molecular states of several tumor entities; however, the real clinical impact of this imaging modality remains to be elucidated further. Therefore, additional randomized controlled trials have to evaluate the effects of hypoxia imaging on the patient outcome.

Beyond imaging glucose consumption and hypoxia, PET ligands targeting the somatostatin receptor (SSR) such as ^68^Ga-DOTATATE can be used for imaging of undifferentiated nasopharyngeal cancer (NPC). Usually, SSR-ligands are used for imaging neuroendocrine tumors and meningioma [38, 71]. In NPC patients, SSR PET provides a high target-to-background contrast, which might be of particular help when infiltration at the skull base might be present [72–74]. In clinical routine of HNC patients, however, ^18^F-FDG-PET/CT plays the key role among these ligands.

Comprehensive response assessment after initial therapy is important as salvage surgery or neck dissection might still be a curative option in these patients. Most problems occur while distinguishing between an incomplete response or inflammatory changes after CRT on conventional imaging. This could be improved by integrating the treatment plan into PET/CT imaging improving and the diagnostic accuracy for response assessment [75]. Moreover, several studies have evaluated the potential use of early treatment response assessment with PET/CT 4 weeks after CRT initiation [76]. In this setting, TLG of the primary tumor is described as prognostic factor for clinical outcome. The intra-therapy reduction of SUV_max_ of the primary tumor was also associated with the locoregional control (LRC) and OS confirming this approach for early response assessment [77, 78]. Of note, PET/CT scans 1 week after CRT show no prognostic value, whereas, by contrast, SUV_max_ of the primary tumor 12 weeks after finalizing therapy [79]. Unnecessary salvage neck dissections can be avoided by response assessment with PET/CT after 12 weeks for node assessment. This was partly validated with correlation to histological specimens [48, 80–82]. Detection of relapse is crucial in the post-treatment care. High false-negative values on conventional imaging can lead to delay the treatment of residual disease and therefore cause worsening in clinical outcome. Hence, ^18^F-FDG PET/CT with its high diagnostic accuracy is indispensable during follow-up [83]. The sensitivity and specificity for the detection of recurrences seems to be the highest between 4 and 6 months after therapy [84–86]. Altogether with the different tracers, high sensitivity and specificity, PET/CT is increasingly finding its way into clinical routine of staging, treatment planning and follow-up of head and neck cancer.

## Lung cancer

Lung cancer remains the leading cause of cancer incidence and death worldwide, with 2.1 million new lung cancer cases and 1.8 million deaths predicted in 2018, corresponding to a fifth of cancer deaths. Non-small cell lung cancer (NSCLC) represents 80–90% of lung cancers, while small cell lung cancer (SCLC) shows a decreasing incidence in many countries over the past two decades [87, 88].

**Fig. 3:**
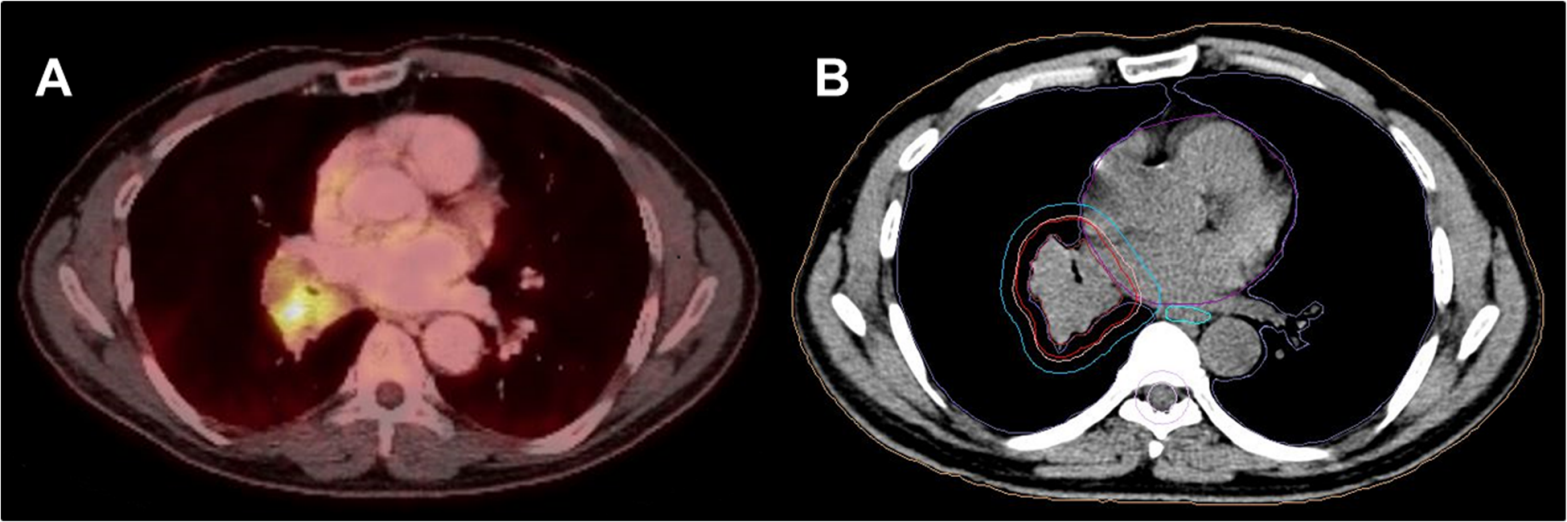
A patient with newly diagnosed NSCLC (cT2b N3 M1b) and ^18^F-FDG PET/CT for staging (**a**) and inclusion in radiotherapy planning (**b**)

PET with ^18^F-FDG is widely used for staging patients with NSCLC [89, 90]. In the PLUS trial, a large multicenter study, patients received either PET/CT staging or only conventional diagnostic CT. Here, the additional use of PET imaging to complete the staging prevented unnecessary surgery in a fifth of patients [91]. Beyond unnecessary surgery, superior mediastinal staging on PET vs. CT was confirmed in a large meta-analysis [92]. Overall, the combined information of hybrid imaging with PET/CT has been shown to have greater staging accuracy than both imaging modalities alone [93–97]. Recently, a number of studies have characterized the diagnostic value of PET/MRI demonstrating an equivalently high diagnostic performance in T and N staging of NSCLC [98–100]. For an example, see Fig. 3.

With regard to radiation treatment planning, ^18^F-FDG PET/CT has proven utility in accurate target volume delineation (TVD) [5]. On ^18^F-FDG PET-CT, delineation of the metabolic tumor volume (MTV) with exclusion of abnormalities e.g. tumor-associated atelectasis or infiltrates improves inter- and intra-reader reproducibility [101, 102]. Thus, combined PET/CT acquisition is the standard method of acquiring ^18^F-FDG PET images for the purposes of baseline staging and for radiation treatment planning [103]. A large systematic review and meta-analysis confirmed that ^18^F-FDG PET/CT for radiation treatment planning in lung cancer has a significant influence on the target definition. Approximately 40% of patients had a significant change in target definition and 20% of patients were no longer eligible for curative intent (chemo)radiotherapy [104].

In 2015, the International Atomic Energy Agency (IAEA) provided a consensus report on PET/CT imaging for TVD in curative intent radiotherapy for NSCLC – herein, extensive recommendations are provided for PET and CT image visualization and interpretation, tumor delineation and using planning CT with and without breathing motion compensation [105].

Furthermore, regarding assessment of treatment response, a secondary analysis of patients with pretreatment and post-induction PET/CT enrolled to the ESPATUE study - a phase 3 study of surgery vs. definitive concurrent CRT boost in patients with resectable stage IIIA[N2] and selected IIIB NSCLC after induction chemotherapy consisting of 3 cycles of cisplatin/paclitaxel and concurrent CRT (1.5 Gy twice-daily plus concurrent cisplatin/vinorelbine) was performed. The percentage of maximum standardized uptake value (%SUV_max_) remaining in the primary tumor after induction chemotherapy (%SUV_remaining_) was predictive of survival and freedom from extracranial progression [106]. This parameter can be used for treatment stratification after induction chemotherapy or for evaluation of adjuvant novel systemic treatment options e.g. immuno-oncology (IO) therapies for high-risk patients. In the practice changing PACIFIC trial, which randomized stage III NSCLC patients to consolidation durvalumab vs. placebo every two weeks for up to 12 months following platinum-based concurrent CRT, data on inclusion of ^18^F-FDG PET/CT in up-front staging and delivery of radiation therapy (pre- vs. post-treatment) is not available and would be of high interest [107, 108]. Currently a number of phase 2 studies are assessing the potential of PD-L1-directed PET imaging e.g. ^89^Zr-durvalumab or ^89^Zr-pembrolizumab to determine SUV of radiolabeled IO uptake in tumor lesions, correlate between tumor uptake and PD-L1 expression as determined by immunohistochemistry and potentially predict response rate (NCT03829007), (NCT03853187), (NCT02760225).

However, the role of PET/CT in SCLC remains uncertain, although international guidelines recommend its use [109]. Evidence has largely been based on small retrospective and non-randomized prospective studies, which have shown improvement in staging accuracy as well as providing additional prognostic information [110–113]. An unplanned post-hoc analysis of patients staged with additional ^18^F-FDG PET/CT (309/540 patients) in the CONVERT study – a multicenter phase 3 study, which randomized patients with limited stage SCLC to twice daily (45 Gy in 30 fractions or once-daily (66 Gy in 33 fractions) platinum-based CRT, survival outcomes (OS, PFS) were not significant. However, patients staged with ^18^F-FDG PET/CT had smaller gross tumor volumes and received lower radiation doses to normal tissue (lung, heart, and esophagus). Caution should be taken, when interpreting these data as inherent confounders cannot be excluded. Importantly, the analysis does not address the role of ^18^F-FDG PET/CT for TVD in particular [114].

Furthermore, there are some drawbacks vis-à-vis response assessment on PET e.g. acquisition time, PET parameters (SUV, total lesion glycolysis (TLG) and MTV) and cut-offs. The lack of an undisputed, univocal parameter remains a challenge. Nevertheless, a current systematic review addressed the role of interim ^18^F-FDG PET/CT during.

CRT for early prediction of clinical outcome in NSCLC and showed that early identification of metabolic tumor response was a predictor of response and prognosis in NSCLC patients [115]. Kong et al. conducted a phase II trial in 42 inoperable stage II-III NSCLC patients, delivering individualized conformal CRT (39 pts./93%) or RT alone (3 pts./7%) to a fixed risk of radiation-induced lung toxicity (grade > 2) and adaptively escalating the dose to the residual tumor defined on mid-treatment FDG-PET after an equivalent dose in 2 Gy per fraction (EQD2) of 50 Gy up to a physical total dose of 86 Gy (EQD2 of 92 Gy [alpha / beta = 10 for tumor]) in 30 daily fractions and showed favorable LRC [116]. The RTOG 1106 trial is a follow-up ongoing clinical study from the same group validating this finding in a randomized manner (NCT01507428).

Moreover, residual metabolic tumor volume after definitive treatment could further determine prognosis: Ohri et al. reported that 30-month cumulative incidence rates of local progression were 32 and 5% for lesions with residual MTV > 25 cm^3^ vs. < 25 cm^3^, respectively [117]. Roengvoraphoj et al. demonstrated that pre-treatment primary tumor (PT)-MTV < 63 cm^3^, post-treatment PT-MV < 25 cm^3^ and ≥ 15% reduction in mid- to post-PT-MV significantly improved OS [118]. In a follow-up analysis, the same group analyzed the prognostic value of a post-treatment PET/CT and demonstrated that PT-MTV reduction of at least 80% (complete and major metabolic response) improved patient outcome [119].^37^

## Prostate cancer

PET/CT PSMA-ligands labeled with ^68^Ga or ^18^F is increasingly used in prostate cancer screening worldwide, as it provides an excellent target-to-background ratio leading to an improved detection rate [120]. PSMA is highly specific for prostatic tumoral tissue, even if PSMA expression in ganglia, sarcoidosis or benign bone diseases may lead to incidentally false-positive findings [121, 122]. A significant benefit in lymph node staging has been observed for PSMA PET/CT compared to standard of care imaging: A recent meta-analysis with histopathology as standard of reference and reported combined sensitivities and specificities of 80 and 97% at lesion level and 86 and 86% at patient level, either at initial staging and/or at biochemical recurrence [123–125]. However, PSMA PET/CT may still underestimate the true extent of nodal involvement, especially with regards to small lymph node metastases < 3 mm [126, 127]. Hence, PSMA PET/CT at the current state may probably not yet allow a perfect node-based therapeutic approach alone, e.g. stereotactic body radiotherapy (SBRT) in comparison to elective nodal radiotherapy (ENRT) [128] or limited salvage lymph node dissection (SLND) compared to super-extended SLND [129]. For detection of bone metastases, PSMA PET/CT outperformed planar bone scans in two large analyses [130, 131]. Only few institutions have the possibility to perform PSMA PET/MRI and head to head comparisons between PSMA PET/MRIs and PET/CT are scarce. Overall, there seems to be a very low discordance between the two imaging techniques including PET-positive lymph nodes of normal size [132].

**Fig. 4:**
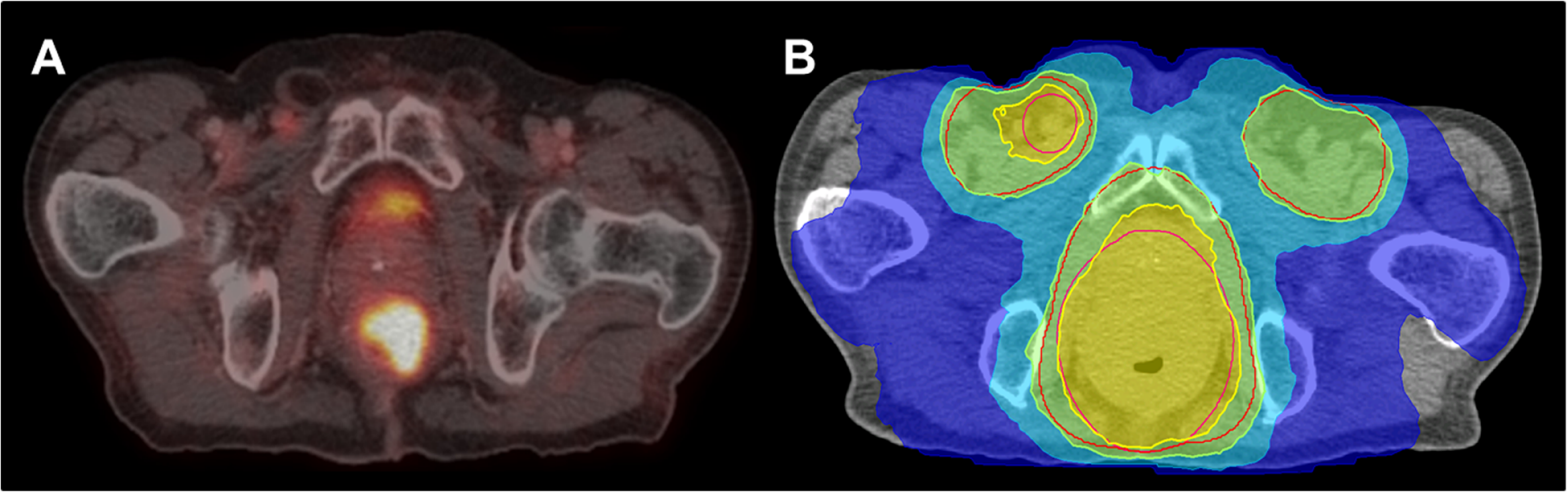
A 68-years old male patient with newly diagnosed anal cancer Stage IIIC (cT4 cN1 cM0) and ^18^F-FDG PET/CT with ^18^F-FDG avid primary tumor and inguinal lymph node (**a**). ^18^F-FDG PET/CT was then used for radiotherapy planning with boost to the right inguinal lymph nodes and primary tumor (**b**)

One further unique characteristic of PSMA PET/CT is its high detection rate of metastases even at low pre-PET PSA levels, e.g. at PSA level < 0.2 ng/ml in 33% and at 0.2–0.49 ng/ml in 45% of the patients, which partly explains the high impact of PSMA PET/CT on the individual patient management, particularly, in patients with biochemical recurrence or persistence [123, 133, 134]. So far, high-level evidence on the benefit of an earlier detection of node or distant metastases is missing and randomized controlled trials evaluating the management and outcome of patients with PSMA PET-positive disease are currently under way. Thus, up to now there are no clear recommendations in the European or NCCN guidelines on application of PSMA PET/CT at initial staging and only a weak recommendation for patients with persistent or recurrent PSA [135]. Nevertheless, particularly in the postoperative setting in persistent or recurrent disease prior to radiotherapy, evidence is accumulating: recently, a single-arm prospective trial on 635 patients with biochemically recurrent prostate cancer reported a high detection rate of 75% and a substantial inter-reader reproducibility for PSMA PET/CT [136]. From a prospective survey, it is known that information from PSMA PET/CT lead to management changes in more than 50% of prostate cancer patients with biochemical recurrence [137]. With special regard to radiotherapy, the specific impact of PSMA PET/CT on salvage radiotherapy was investigated in patients with a PSA recurrence of < 1.0 ng/ml after radical prostatectomy: In this analysis, patients had a median PSA of 0.48 ng/ml and the PSMA PET/CT result was compared to standard of care Radiation Therapy Oncology Group (RTOG) clinical target volume (CTV) of both the prostate bed and the pelvic lymph nodes. Overall, 132 of 270 included patients had PSMA PET-positive lesions and 52 patients had at least one lesion not covered by consensus CTVs [138]. These findings have led to a randomized imaging trial of salvage radiotherapy with or without PSMA PET/CT (NCT03582774) investigating its potential benefit on clinical outcome in a prospective setting [139]. So far, few retrospective studies reported patient outcome after PSMA PET/CT-based salvage radiotherapy. The mean PSA response rate in these studies was 74% (range, 60–83%) after a mean follow-up time of 19 months [140–144], for an example see Fig. 4. Overall, one might expect that PSMA PET/CT improves salvage radiotherapy and thereby potentially outcome in numerous ways: Firstly, visualizing macroscopic recurrence allows for dose-escalation. Secondly, CTVs can be expanded to areas not normally treated by consensus CTV. Thirdly, macroscopic recurrence might lead to early application of androgen deprivation therapy concurrent with radiotherapy and finally, salvage radiotherapy might be abandoned in case of overt metastatic disease. Regarding primary prostate cancer, current research is focusing on the identification and accurate contouring of the intraprostatic tumor volume based on PSMA PET/CT in order to allow for focal radiation therapy with dose escalation to the PSMA PET-positive lesions within the prostatic gland [145, 146]. For intraprostatic boosting, PSMA PET/CT may even replace multiparametric MRI (mpMRI): In a prospective validation study, an increased consensus of PSMA PET/CT with histopathologic correlation was observed for intraprostatic gross tumor volume delineation compared to mpMRI. Additionally, mpMRI contours significantly underestimated tumor volume [147]. Also, analysis of radiomic features gain access into the evaluation of prostate cancer patents [148]. In recent years, treatment of oligometastatic prostate cancer has sparked new interest since the STAMPEDE trial reported a significant overall survival benefit by prostate radiotherapy and life-long ADT in patients with low metastatic burden [149] and the STOMP trial an ADT-free longer survival by metastasis-directed therapy vs. surveillance [150]. This has led to various studies all incorporating PSMA PET/CT as diagnostic imaging like the PEACE V trial (NCT03569241) or the “Prostate Cancer Subclinical Metastatic Ablative MR-guided Radiotherapy” study (NCT03160794).

Moreover, in analogy to neuroendocrine tumors, where radioligands labeled with the beta-emitters such as ^177^Lu-DOTATATE are approved and successfully used in clinical routine [151], PSMA-ligands labeled with ^177^Lu can also be used for radioligand therapy [152]. In several countries, PSMA-ligands show to be a valuable treatment option in patients with metastasized, castration-resistant prostate cancer, so that several trials such as the “VISION” trial (NCT03511664) are on their way.

Overall, the use of PSMA PET/CT prior to radiotherapy of primary or postoperative prostate cancer warrants further high-level research to find its rightful place in the current guidelines. Nevertheless, the available evidence already suggests that PSMA PET/CT will become an even more decisive tool in the guidance and treatment of prostate cancer patients than it nowadays already is.

## Gastrointestinal oncology

Radiation oncology is a crucial part of the treatment in several gastrointestinal tumors. Particularly in curative intended treatment of esophageal carcinoma, rectal cancer, and anal cancer radiotherapy (RT) is a key treatment modality. The impact of ^18^F-FDG PET/CT on staging of patients with gastrointestinal malignancies, radiation treatment planning, and response assessment is well established.

### Esophageal cancer

In curative treatment of patients with locally advanced esophageal cancer chemoradiation (CRT) is either performed as neoadjuvant treatment before surgery or as definitive treatment in case of unresectable tumor. Neoadjuvant treatment is performed in patients who are fit to undergo major surgery. However, patients with thoracic squamous cell carcinoma of the esophagus who show good response to CRT might have a similar outcome with definitive CRT compared to surgery [153]. Several studies have gathered evidence that ^18^F-FDG PET/CT has a high prognostic value in patients undergoing CRT for esophageal cancer [154, 155] and can therefore be useful to guide treatment decisions.

RT planning in patients with esophageal cancer is challenging since the primary tumor is often poorly visible on standard morphological imaging with CT alone. Given the submucosal spread of the tumor, diagnostic modalities such as barium swallow, gastroesophagoscopy or MRI are frequently used to further determine the exact tumor extend. While the additional effect of ^18^F-FDG PET/CT on primary tumor volume delineation seems limited with different studies reporting conflicting results either with benefit [156] or no benefit [157] of PET/CT over conventional imaging alone. In contrast, the effect on metabolic imaging on the identification of involved nodes has been shown [157, 158]. ^18^F-FDG PET/CT has a high specificity and sensitivity in detecting involved nodes in esophageal cancer and should therefore be considered for pre-treatment imaging in patients with esophageal cancer [159].

### Pancreatic cancer

RCT or stereotactic radiotherapy [160] can be offered to patients as part of multidisciplinary treatment in locally advanced pancreatic cancer (LAPC) either as neoadjuvant or as definitive treatment. The pancreas is localized in close proximity to highly radiation sensitive organs such as the duodenum and small bowel that need to be spared [161]. Large safety margins to account for internal movement and positioning uncertainties are therefore obsolete and precise tumor delineation remains the main challenge in treatment planning of LAPC. While malignancies of the pancreas can be imaged using different PET tracer such as ^18^F-FDG and ^18^F-fluorothymidine (FLT) [162] the value of metabolic imaging for delineating GTV in pancreatic cancer remains debatable. Due to a long acquisition phase encompassing several breathing cycles misregistration between PET and CT occur and can lead to uncertainties in determining the actual tumor extend [163].

Concerning patient management pre-therapeutic ^18^F-FDG PET/CT has a prognostic value as initial low SUV_max_ showed an association with better median survival after SBRT in one study with 55 LAPC patients [164]. Accordingly another study with 33 patients reported that high metabolic tumor volume (MTV) and total lesion glycolysis (TLG) prior to induction gemcitabine and SBRT were associated with inferior overall survival [165]. A histopathological correlation in patients with borderline resectable pancreatic carcinoma and LAPC undergoing multimodality neoadjuvant treatment showed both post-neoadjuvant CA19–9 and post-neoadjuvant therapy SUV_max_ significantly correlating with tumor regression grade [166].

### Rectal cancer

Neoadjuvant RT or CRT before total mesorectal excision (TME) is well established in the treatment of locally advanced rectal cancer (LARC). It has been proven that pathological complete response (pCR) after CRT correlates with improved long-term outcome [167]. Moreover, a correlation between applied radiation dose and pCR was found [168, 169]. Therefore, to further improve response to neoadjuvant treatment while sparing normal tissue focal dose escalation on macroscopic tumor seems worthwhile [170]. Commonly, CT-based radiation treatment planning is complemented by MRI information for better soft tissue contrast. In a prospective study, gross tumor volume delineation on MRI and ^18^F-FDG PET/CT was compared in 77 patients [171]. The authors concluded that ^18^F-FDG PET/CT added important information for the delineation process with PET-based GTV been smaller than MRI-based GTV but larger GTV volumes when both MRI and PET information was used. A comparison of tumor extend in pathological specimens even showed a better correlation to tumor extend measured on ^18^F-FDG PET/CT than on CT or MRI [172] underlining the importance of including metabolic imaging in the initial treatment planning.

In up to 20% of patients with LARC pCR can be achieved after neoadjuvant CRT [173]. This high rate of pathological response fuels efforts to provide organ-preserving treatment to patients with good clinical response [174] thus emphasizing the need for valid treatment assessment. So far, post-treatment ^18^F-FDG PET/CT has been evaluated extensively in retrospective studies with promising results [175–178]. However, at this stage, further validation is needed.

### Anal cancer

Squamous cell carcinoma of the anus (SCCA) is a rare disease accounting for approximately 1–2% of gastrointestinal tumors only [179]. CRT is the treatment of choice for curative intended organ-preserving treatment with surgery reserved for salvage. The majority of SCCA is highly ^18^F-FDG-avid [180] and multiple studies have described a change in tumor staging in up to 20% of cases with the use of ^18^F-FDG PET/CT [181, 182].

**Fig. 5:**
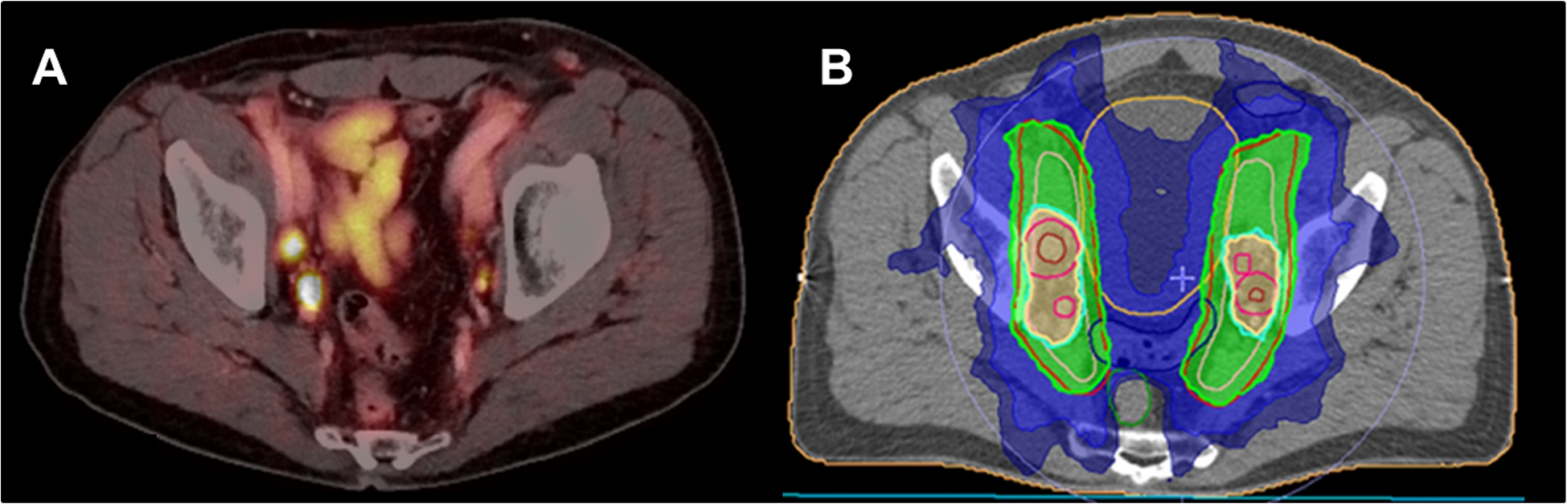
A 69-years old patient with biochemical recurrence of prostate cancer (pT2c pN0 R0 Gleason score 9, preoperative PSA 11.7 ng/ml) and evidence of PET-positive lymph node metastases (**a**) and radiotherapy-plan with dose-escalation to the PET-positive lymph nodes (**b**)

Concerning radiation treatment planning metabolic imaging can be useful to guide target definition of elective lymph nodes regions and boost to primary tumor, see Fig. 5. A recent meta-analysis reported a change in target volume delineation with the use of ^18^F-FDG PET/CT in almost 25% of patients [183] compared to treatment planning based on conventional imaging. More specifically, ^18^F-FDG PET/CT contributes to identifying involved lymph nodes that need to be included into the radiation field. A recent study evaluated the distribution of involved lymph nodes on PET/CT and correlated their findings with three established delineation guidelines. The authors reported detection of involved lymph nodes outside the borders of standardized clinical target volumes (CTV) recommended by the respective delineation guidelines of 10–29% [184]. The impact on ^18^F-FDG PET/CT on delineation of primary tumor gross tumor volume (GTV) has been described by Krengli et al. They reported that metabolic imaging had a greater influence on GTV definition than on CTV delineation [185]. Attempts to identify ^18^F-FDG-avid sub-volumes for further dose escalations within the primary tumor have been made [186] however, this approach needs further research. Utilization of metabolic imaging as biomarker for the prediction of treatment outcome in anal cancer has been evaluated intensively. Several retrospective and prospective studies have reported on the value of ^18^F-FDG PET and PET/CT for early detection of tumor recurrence as well as the predictive value of different parameters in pre- and posttreatment ^18^F-FDG PET/CT for response assessment [187–190] with promising results.

## Summary

PET imaging is increasingly used in the clinical management of patients undergoing radiotherapy. Especially, this is the case for a broad variety of cancer entities, as presented in the current manuscript. Moreover, PET imaging is increasingly included in randomized trials focused on radiotherapy, where parameters from PET are used as imaging biomarkers. Hence, in radiation oncology practice, imaging biomarkers derived from PET comprise valuable additional information beyond standard morphologic imaging for tumor staging, radiotherapy planning, but also - after undergoing radiotherapy - for treatment monitoring and the differentiation of tumor relapse from inflammatory or radiation induced changes. So far, ^18^F-FDG is the most widely used tracer as described above; however, there is a strong trend towards radioligands, which target more specific tumor features rather than only glucose metabolism, e.g. PSMA-targeted ligands for prostate cancer imaging or SSR-directed ligands for meningioma imaging. Along with anticipated technical innovations such as whole body PET, methodological innovations such as the application of PET-derived radiomics and deep learning methods will further improve tumor characterization, identification and, hence, the clinical workup of patients undergoing radiotherapy.

## Data Availability

Data sharing not applicable to this article as no datasets were generated or analysed during the current study.
